# PPAR*α* in Obesity: Sex Difference and Estrogen Involvement

**DOI:** 10.1155/2010/584296

**Published:** 2010-08-17

**Authors:** Michung Yoon

**Affiliations:** Department of Life Sciences, Mokwon University, Taejon 302-729, Republic of Korea

## Abstract

Peroxisome proliferator-activated receptor *α* (PPAR*α*) is a member of the steroid hormone receptor superfamily and is well known to act as the molecular target for lipid-lowering drugs of the fibrate family. At the molecular level, PPAR*α* regulates the transcription of a number of genes critical for lipid and lipoprotein metabolism. PPAR*α* activators are further shown to reduce body weight gain and adiposity, at least in part, due to the increase of hepatic fatty acid oxidation and the decrease in levels of circulating triglycerides responsible for adipose cell hypertrophy and hyperplasia. However, these effects of the PPAR*α* ligand fenofibrate on obesity are regulated with sexual dimorphism and seem to be influenced by the presence of functioning ovaries, suggesting the involvement of ovarian steroids in the control of obesity by PPAR*α*. In female ovariectomized mice, 17*β*-estradiol inhibits the actions of fenofibrate on obesity through its suppressive effects on the expression of PPAR*α* target genes, and these processes may be mediated by inhibiting the coactivator recruitment of PPAR*α*. Thus, it is likely that PPAR*α* functions on obesity may be enhanced in estrogen-deficient states.

## 1. Introduction

Obesity is the result of an energy imbalance caused by an increased ratio of caloric intake to energy expenditure. In conjunction with obesity, related metabolic disorders such as dyslipidemia, atherosclerosis, and type 2 diabetes have become global health problems. The peroxisome proliferator-activated receptors (PPARs) have been the subject of intense investigation and considerable pharmacological research due to the fact that they are involved in the improvement of these chronic diseases. Three PPAR isotypes have been identified: PPAR*α*, PPAR*γ*, and PPAR*β*/*δ*, each with different ligand specificity, very distinct tissue distributions, and different biological functions. 

Among the three subtypes, PPAR*α* is expressed predominantly in tissues that have a high level of fatty acid (FA) catabolism such as liver, heart, and muscle [1–3]. PPAR*α* regulates the expression of a large number of genes that affect lipid and lipoprotein metabolism [4–7]. PPAR*α* ligands fibrates have been used for the treatment of dyslipidemia due to their ability to lower plasma triglyceride levels and elevate HDL cholesterol levels. PPAR*α* is also thought to be involved in energy metabolism. Since PPAR*α* ligands fibrates stimulate hepatic FA oxidation and thus reduce the levels of plasma triglycerides responsible for adipose cell hypertrophy and hyperplasia, PPAR*α* may be important in the control of adiposity and body weight due to its ability to regulate an overall energy balance. This notion is supported by findings showing that PPAR*α*-deficient mice exhibited abnormalities in triglyceride and cholesterol metabolism and became obese with age [8]. Furthermore, several studies have suggested that fibrates can modulate body weight and adiposity in experimental animal models, such as fatty Zucker rats, high fat-fed C57BL/6 mice, and high fat-fed obese rats [9–11].

Energy balance seems to be influenced by gonadal sex steroids [12]. Female sex steroid hormones have been the subject of intense investigation over the last several decades based on the role that these ovarian hormones play in regulating food intake, body weight, and lipid metabolism. For example, ovariectomized (OVX) animals and postmenopausal women show increased food intake, body weight, and adipose tissue mass, as well as decreased FA oxidation and triglyceride lipolysis, indicating the involvement of gonadal steroids in the modulation of obesity [13–16]. Several lines of study show that ovarian steroids, in particular estrogens, can affect obesity and the related disorders of dyslipidemia, type 2 diabetes, and cardiovascular disease (CVD) [12]. Estrogen insufficiency is known to be largely responsible for increased adiposity and circulating lipids in OVX rodents because such animals do not display obesity, adiposity, and lipid disorders when they are administered exogenous estrogens [17–19]. Moreover, my previous results demonstrated that fenofibrate reduced body weight and white adipose tissue (WAT) mass in male and female OVX mice [20–23]. Although the administration of 17*β*-estradiol (E2) or fenofibrate alone effectively reduces body weight gain and WAT mass in female OVX mice, fenofibrate treatment does not prevent gains in body weight and WAT mass in the presence of ovaries. Interestingly, there are data indicating that PPAR/RXR heterodimers are capable of binding to estrogen response elements (EREs), and PPAR and estrogen receptors (ERs) share cofactors [24–28], suggesting that signal cross-talk may exist between PPAR*α* and ERs in the control of obesity. 

Based on my published results showing the fenofibrate functions on obesity during various conditions, this paper will focus on the differential regulation of PPAR*α* on obesity by sex differences and the interaction of PPAR*α* and ERs in the regulation of obesity.

## 2. General Aspects of PPAR*α* and ERs

### 2.1. PPAR*α* and ERs as Nuclear Hormone Receptors

**Figure 1:**
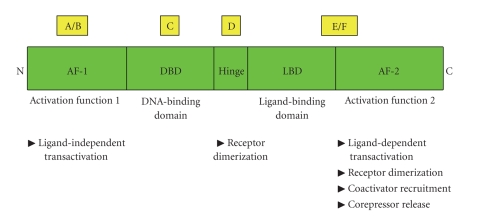
*Schematic structure of the functional domains of nuclear receptors.* The activation domains AF-1 and AF-2 are located at the N-terminal and C-terminal regions, respectively. C domain is a highly conserved DNA-binding domain. D domain is a highly flexible hinge region. E/E domain is responsible for ligand-binding and converting nuclear receptors to active forms that bind DNA. Adapted from [29].

Both PPAR*α* and ERs belong to the nuclear hormone receptor superfamily, which has a typical structure consisting of six functional domains, A/B, C, D, and E/F (Figure 1) [29–31]. The amino-terminal A/B domain contains a ligand-independent activation function-1 (AF-1). The C or DNA binding domain (DBD) contains the structure of the two zinc fingers and *α*-helical DNA motifs. The DBD directs nuclear receptors to the hormone response elements (HREs) of target genes. The D region is a highly flexible hinge region and may be involved in protein-protein interactions, such as receptor dimerization and efficient binding of DBD to HREs. The E/F domain is responsible for ligand-binding and is thus named the ligand binding domain (LBD). The interaction of nuclear receptors with their ligands induces conformational changes that include the AF-2 ligand-dependent activation domain, which is located in the C-terminal *α*-helix. AF-2 regulates ligand-dependent transactivation, recruitment of coactivators, and release of corepressors. In addition, AF-2 is also important for receptor dimerization. 

**Figure 2:**
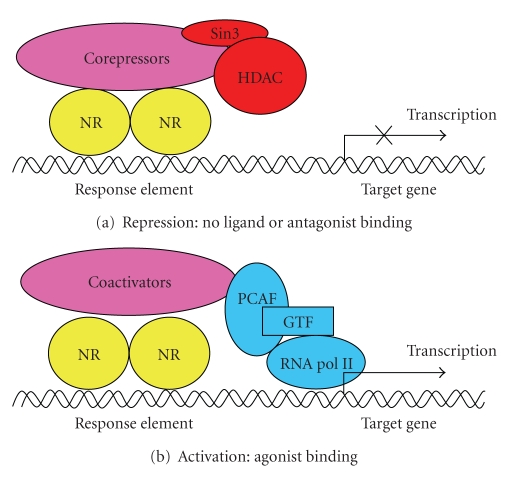
*Activation and repression of nuclear receptor activity.* (a) In the absence of ligand, nuclear receptors (NRs) are associated with corepressor complexes that bind Sin3 and histone deacetylase (HDAC), thereby turning off gene transcription. Some steroid receptors can recruit this complex when they are occupied by antagonists although they do not seem to be associated with corepressors in the unliganded state. (b) In the presence of ligand, NRs generally recruit coactivator complexes, PCAF histone acetyltransferase protein, general transcription factors, and RNA polymerase II to induce gene transcription. GTF: general transcription factor; RNA pol II: RNA polymerase II; PCAF: P300/CBP-associated factor.

Molecular signaling of PPAR*α* and ERs functions is similar [34–37]. In the unliganded or antagonist-bound state, they are associated with corepressor proteins such as nuclear receptor corepressor (NCoR) or silencing mediator of retinoic acid and thyroid hormone receptor (SMRT) (Figure 2(a)). After binding within the LBD, PPAR*α* ligands induce heterodimerization with retinoid X receptor (RXR) and the subsequent interaction with coactivators like CREB-binding protein (CBP) or steroid receptor coactivators, followed by binding to PPAR response elements (PPREs) within target gene promoters (Figure 2(b)). Similarly, ligand-activated ERs bind to their half-site-containing EREs as homodimers following the recruitment of coactivators. Importantly, PPAR*α* shares a similar pool of cofactors with ERs which provides a basis for mutual interactions between these receptors [34, 35]. 

### 2.2. PPAR*α*

PPAR*α* was the first PPAR to be identified by Issemann and Green in 1990, and human PPAR*α* was cloned by Sher et al. in 1993 [1, 38]. PPAR*α* is predominantly expressed in tissues with high rates for mitochondrial and peroxisomal FA catabolism such as liver, brown adipose tissue (BAT), heart, skeletal muscle, kidney, and intestinal mucosa [1–3]. Significant amounts of PPAR*α* are present in different immunological and vascular wall cell types [39, 40]. 

PPAR*α* acts as a ligand-activated transcription factor. PPAR*α* mediates the physiological and pharmacological signaling of synthetic or endogenous PPAR*α* ligands. FAs and FA-derived compounds are natural ligands for PPAR*α*. Modified FAs, conjugated FAs, oxidized phospholipids, and FA-derived eicosanoids such as 8-*S*-hydroxytetraenoic acid and leukotriene B4 activate PPAR*α* [41]. Synthetic compounds can also activate PPAR*α*. These compounds include carbaprostacyclin, nonsteroidal anti-inflammatory drugs, pirinixic acid (also known as Wy14,643), phthalate ester plasticizers, and hypolipidemic drugs fibrates [41]. Of the currently used fibrates, fenofibrate, gemfibrozil, clofibrate, and ciprofibrate preferentially activate PPAR*α* whereas bezafibrate activates all three PPARs. Novel PPAR*α*/*γ* dual agonists and PPAR*α*/*γ*/*δ* pan agonists with PPAR selective modulator activity are under development as drug candidates [42, 43].

**Figure 3:**
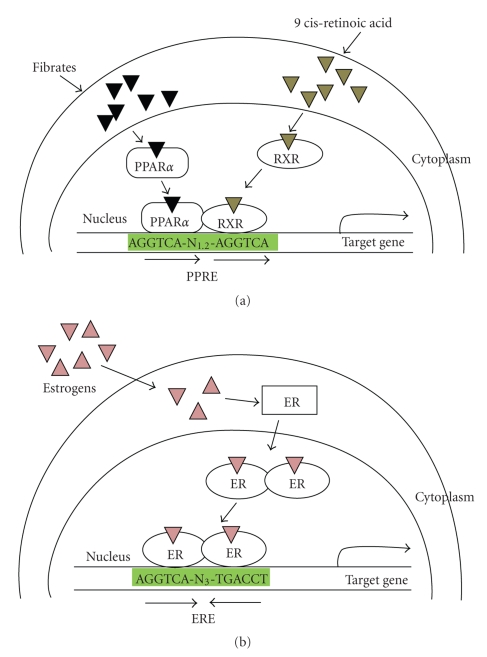
*The signaling pathways of* *P* *P* *A* *R* *α* *and estrogen receptors.* (a) After activation by its respective ligands, PPAR*α* heterodimerizes with retinoid X receptor and binds to direct repeat PPRE in the promoters of target genes to drive expression of target genes. (b) Estrogen-bound estrogen receptors recognize palindromic ERE to directly bind this DNA and ultimately increase gene expression. RXR: retinoid X receptor; PPRE: PPAR response element; ERE: estrogen response element; ERs: estrogen receptors.

PPAR*α* regulates the expression of a number of genes critical for lipid and lipoprotein metabolism, thereby leading to lipid homeostasis. Ligand-bound PPAR*α* heterodimerizes with RXR and binds to direct repeat PPREs in the promoter region of target genes (Figure 3(a)). PPAR*α* target genes include those involved in the hydrolysis of plasma triglycerides, FA uptake and binding, and FA *β*–oxidation (Table 1). Genes involved in the HDL metabolism are also regulated by PPAR*α*. The activation of PPAR*α* target genes therefore promotes increased *β*-oxidation of FAs, as well as the decrease in high circulating triglyceride levels and increased high HDL cholesterol levels, leading to lipid homeostasis.

In addition to PPAR*α* regulation of genes for lipid and lipoprotein metabolism, PPAR*α* regulates the expression of uncoupling proteins (UCPs), which contain PPRE in their promoters. PPAR*α* activators increase the mRNA levels of UCP1 in BAT, UCP2 in liver, and UCP3 in skeletal muscle. UCP1 regulates energy expenditure through thermogenesis. Reductions in body weight and adiposity by fenofibrate are associated with elevation of hepatic UCP2 expression [44]. Transgenic mice overexpressing UCP3 in their skeletal muscle exhibit increased FA oxidation and are resistant to diet-induced obesity. Thus, PPAR*α* may be involved in energy balance and obesity by regulating UCPs [45].

In addition to the important roles of PPAR*α* in FA oxidation in liver and skeletal muscle, PPAR*α* activators may affect adipose tissue metabolism. For example, administration of bezafibrate, a typical PPAR activator, leads to dedifferentiation of adipocytes into preadipocyte-like cells through the activation of genes involved in both mitochondrial and peroxisomal *β*-oxidation [46]. The PPAR*α* ligand GI259578A decreases the mean size of adipocytes in WAT [47]. This is supported by my recent report that fenofibrate stimulates FA *β*–oxidation in both epididymal adipose tissue and differentiated 3T3-L1 adipocytes [48]. 

PPAR*α* may be involved in the regulation of energy balance through fat catabolism. Since fenofibrate increases hepatic FA oxidation and thus decreases the levels of plasma triglycerides responsible for adipose cell hypertrophy and hyperplasia, it may inhibit an increase in body weight. This is supported by a report that PPAR*α*-deficient mice showed abnormal triglyceride and cholesterol metabolism and became obese with age [8]. Expression of PPAR*α* and FA oxidative PPAR*α* target genes is suppressed in obese mice [49]. Many studies show that fenofibrate can modulate body weight in animal models of diabetes, obesity, and insulin resistance although another known PPAR*α* stimulator perfluorooctanoic acid induces overweight at low doses in intact female mice [9–11, 50]. 

PPAR*α* also regulates insulin resistance and diabetes due to visceral obesity. Fenofibrate prevents adipocyte hypertrophy and insulin resistance by increasing FA *β*-oxidation and intracellular lipolysis from visceral adipose tissue, showing that PPAR*α* may be one of the major factors leading to decreased adipocyte size and improved insulin sensitivity [48]. Moreover, PPAR*α* agonist treatment has been reported to improve pancreatic *β*-cell function in insulin-resistant rodents and the adaptive response of the pancreatic *β*-cell function to pathological conditions, such as obesity [51, 52]. In addition, PPAR*α* agonists, including fibrates, normalize atherogenic lipid profile, as well as several cardiovascular risk markers [53].

### 2.3. ERs

Like PPAR*α*, ERs function as ligand-dependent transcription factors belonging to members of the nuclear hormone receptor family. Two major ERs (ER*α* and ER*β*) mediate the physiological and pharmacological signals of natural or synthetic ER activators. Upon estrogen binding, ERs are activated and act as transcriptional modulators by binding to palindromic EREs in the promoter region of target genes (Figure 3(b)) [54, 55]. ERs are also activated by specific synthetic ligands such as raloxifene, tamoxifen, and the ER*β*-specific ligand diarylpropionitrile. ER*α* is mainly expressed in the female reproductive system such as ovary, uterus, pituitary, and mammary glands but is also present in the hypothalamus, brain, bone, liver, WAT, skeletal muscle, and the cardiovascular system [56–58]. ER*β* is expressed in many tissues including skeletal muscle, WAT, BAT, prostate, salivary glands, testis, ovary, vascular endothelium, the immune system, and certain neurons of the central and peripheral nervous system [59, 60]. 

The natural forms of estrogens are E2, estrone, and estriol. E2 potently activates ER-mediated transcriptional activity to a greater extent than estrone or estriol. E2 has been considered one of the most important hormones in female physiology and reproduction for a long period . However, we now know that E2 also plays a protective role in a variety of pathophysiological states, such as obesity, cardiovascular disease, hyperlipidemia, diabetes, osteoporosis, and cancer in both men and women [61].

 E2 is involved in the regulation of adiposity and obesity, and visceral fat varies inversely with E2 levels [62]. Accumulation of visceral fat occurs in females when E2 levels become sufficiently low. In rodents, ovariectomy leads to weight gain primarily in the form of adipose tissue, which is reversed by physiologic E2 replacement [12, 63–65]. Loss of circulating E2 is associated with an increase in adiposity during menopause whereas postmenopausal women who receive E2 replacement therapy do not display the characteristic abdominal weight gain pattern usually associated with menopause [13–15]. Aromatase deficiency, during which E2 is not produced, results in the development of adiposity and obesity [66]. Furthermore, ER*α* deficiency increased adipose tissue in both male and female mice, consistent with other reports linking estrogen with body weight regulation and adipocyte function [67]. E2 influences food intake and eventually the maintenance of normal body weight in adult females. In female dogs, a phasic decrease in food intake occurs during estrus [68]. Gradual decreases in eating through the follicular phase have been shown in monkeys, which show progressive increases in estrogens through the follicular phase comparable to those of humans [69]. E2 treatment to OVX rats normalized meal size, food intake, and body weight gain to the levels observed in intact rats [19, 70]. ER*β* is involved in the anorectic action of E2. Blockade of ER*β* inhibits the effects of E2 on food intake, body weight gain, and fat accumulation in OVX rats [71]. In contrast, Heine et al. [67] and D'Eon et al. [16] suggested that E2 decreases adiposity and adipocyte size in OVX mice independent of differences in energy intake, possibly through promoting fat oxidation and enhancing triglyceride breakdown [16, 67]. 

 In addition to food intake and body weight regulation, estrogen improves glucose homeostasis and diabetes mellitus. Mice that lack ER*α* have insulin resistance and impaired glucose tolerance [67]. Both male and female aromatase-KO mice have reduced glucose oxidation, and male aromatase-KO mice develop glucose and insulin resistance that can be reversed by E2 treatment [58, 66]. ER*α* and ER*β* modulate glucose transporter 4 expression and stimulate glucose uptake in skeletal muscle of mice [58]. Estrogens have also been shown to regulate vascular disease. Premenopausal women have a lower tendency to develop hypertension than do men of similar age, but the prevalence of CVD increases more rapidly in aging women than in men [72]. The increased incidence of CVD in aged women may be due to the development of obesity. Although the rate of increase of CVD is greater at the postmenopausal age in women than at the same age in men, the actual incidence of CVD is still less in women than in men if hypertension is not included (Framington Heart Study). Thus, estrogen signaling through ERs leads to improvement of metabolic disorders.

As mentioned above, both PPAR*α* and ERs have similar structures, action mechanisms, and functions, suggesting the interaction of PPAR*α* with ERs in the control of these metabolic diseases including obesity. However, signal cross-talk between PPAR*α* and ERs in the regulation of obesity is not clear.

## 3. PPAR*α* Functions on Obesity

Over the last several decades, a number of studies have been published on the physiology, pharmacology, and functional genomics of PPAR*α*. In vivo and in vitro studies demonstrate that PPAR*α* plays a central role in lipid and lipoprotein metabolism, and thereby decreases dyslipidemia associated with metabolic syndrome. Obesity is the leading cause for the development of metabolic diseases, such as obesity, type 2 diabetes, dyslipidemia, and CVD. There are important sex differences in the prevalence of obesity-related metabolic diseases [33, 73–75]. Ovarian hormones seem to have protective roles in metabolic diseases since women with functioning ovaries have much fewer incidences of such disorders, but these metabolic diseases dramatically increase in postmenopausal women. 

### 3.1. Fenofibrate Regulates Obesity with Sexual Dimorphism

**Figure 4:**
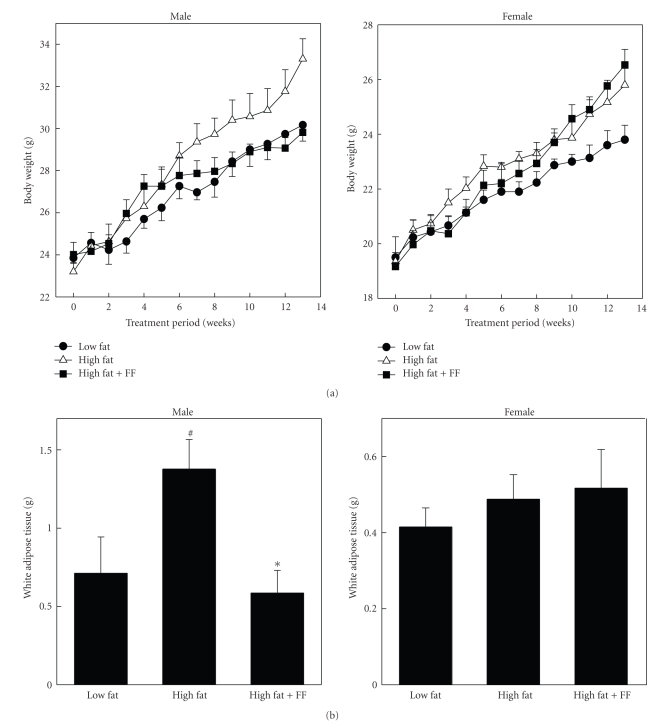
*Effects of fenofibrate on high fat diet-induced body weight gain (a) and WAT mass (b) in both sexes of C57BL/6 mice.* Male and female C57BL/6 mice were received a low fat, high fat, or high fat diet supplemented with fenofibrate (0.05% w/w) for 13 weeks. Body weight at the end of the experiment are statistically different (*P* < .01) between high fat diet and high fat plus fenofibrate groups. #: Significantly different versus a low fat diet group, *P* < .05. ∗: Significantly different versus a high fat diet group, *P* < .01. Adapted from [20].

PPAR*α* activator fenofibrate differentially influences body weight and adiposity in both sexes of mice. Fenofibrate improves body weight gain and adiposity in high fat-diet-fed male mice, but fails to regulate them in female mice (Figure 4) [20]. In males, body weight and WAT mass increased by 44% and 77%, respectively, after 14-week administration of high fat diet. These parameters were lowered after fenofibrate treatment, more so than those of mice given a low fat diet, and the reduction in body weight correlated with a fall in adipose tissue mass. In contrast to males, fenofibrate slightly increased high fat diet-induced body weight and adipose tissue mass in female mice, suggesting a different PPAR*α* action on females than on males in the control of obesity. Previous studies showed that fenofibrate can modulate body weight and adiposity in several animal models [9–11]. Since these results were obtained from males, fenofibrate may be an effective regulator of energy homeostasis in the male animal system. Taken together, these studies show that body weight gain and adipose tissue mass of male C57BL/6 mice were significantly reduced by fenofibrate, but those of females were not, and indicate that the action of fenofibrate on body weight and adiposity is different, depending on sex. 

Although fibrates are drugs widely used to lower elevated plasma triglycerides and cholesterol, fenofibrate is shown to control lipid metabolism with sexual dimorphism. Serum concentrations of total cholesterol and triglycerides were significantly reduced by fenofibrate in male mice, similar to the previous reports [76, 77]. However, fenofibrate not only failed to decrease total cholesterol, but also decreased circulating level of triglycerides in female mice to a much lower extent than in similarly treated males. Based on the information that lipids accumulated in the adipose tissue are largely derived from circulating triglycerides, differential regulation of adiposity by fenofibrate is partly due to different levels of circulating lipids between sexes. 

The regulatory effect of fenofibrate on obesity is not mediated through leptin since PPAR*α*-knockout mice that become obese with age are not hyperphagic [8, 10]. Instead, many reports indicate that fenofibrate-regulated increases in hepatic *β*-oxidation are involved in this process. FA oxidation results in a decrease in FAs available for triglyceride synthesis [78, 79]. According to Yoon et al. [20], fenofibrate elevated the transcriptional activation of PPAR*α* target genes, acyl-CoA oxidase (ACOX), enoyl-CoA hydratase/3-hydroxyacyl-CoA dehydrogenase (HD), and thiolase in both sexes of mice [20]. However, the expression levels were much higher in males than in females, suggesting that fenofibrate exhibits sexually dimorphic activation of PPAR*α* actions on hepatic *β*-oxidation, resulting in the differential energy balance with sex. 

Mancini et al. [11] and Guerre-Millo et al. [10] report that fenofibrate improves obesity due to its action on FA *β*-oxidation in the liver and seems to act as a weight-stabilizer through its effect on liver metabolism [10, 11]. Moreover, the body weights of PPAR*α*-deficient mice were greater than those of wild-type mice, and a marked increased amount of intra-abdominal adipose tissue was seen in PPAR*α*-KO mice. In addition, Costet et al. [8] suggested the involvement of PPAR*α* with a sexually dimorphic control of circulating lipids, fat storage, and obesity, in a study using male and female PPAR*α*-null mice [8]. In contrast to these investigators, Akiyama et al. [80] provided evidence that PPAR*α* regulates lipid metabolism but is not associated with obesity [80]. Similar to the results of Akiyama et al. [80], Yoon et al. [20] provided evidence that fenofibrate is involved in obesity, but not likely to have an effect on obesity mainly through PPAR*α*-mediated action since it increases FA *β*-oxidation and decreases serum triglycerides in female mice, although their effects are much lower compared with males [20]. 

 Overall, fenofibrate treatment affects body weight, adipose tissue mass, lipid metabolism, and hepatic *β*-oxidation with sexual dimorphism, but fenofibrate-regulated obesity is not directly associated with PPAR*α*-mediated action and may be influenced by sex-related factors.

### 3.2. Fenofibrate Improves Male Obesity

Fenofibrate seems to suppress diet-induced obesity and severe hypertriglyceridemia caused by LDL receptor (LDLR) deficiency in male mice. The loss of LDLR increases susceptibility to diet-induced obesity and hypertriglyceridemia. Body weights and WAT mass increased in LDLR-null mice on a high fat diet compared with low fat diet controls [22, 81]. However, fenofibrate prevented the high fat diet-induced increases in body weight and WAT mass in male LDLR-null mice. The body weights of male LDLR-null mice were significantly reduced after 1 week of fenofibrate administration whereas wild-type mice showed weight decreases after 7 weeks of fenofibrate [20, 22], indicating that fenofibrate more effectively reduces body weight gain in LDLR-null mice than in wild-type mice. Interestingly, the final body weight of the fenofibrate-treated obese animals was very similar to that of lean animals on a lowfat diet. High fat diet-fed LDLR-null mice showed hepatic lipid accumulation, which was absent in the hepatocytes of mice on a low fat diet and which disappeared following fenofibrate treatment, mainly due to peroxisomal and mitochondrial *β*-oxidation of FAs [82, 83]. This indicates not only the prevention of body weight gain and the increased fat mobilization from WAT due to fenofibrate-induced increases of fat catabolism in the liver, but also a strong correlation between reduced body weight and decreased WAT mass by fenofibrate. In addition, fenofibrate did not affect food intake in high fat diet-induced obese LDLR-null mice. These results suggest that the increased liver activity may be paralleled by a large reduction in WAT mass, which accounts for most of the body weight reduction. 

Fenofibrate also substantially decreased the increases in circulating triglycerides and total cholesterol levels, indicating that fenofibrate efficiently regulates triglyceride and cholesterol metabolism in male LDLR-null mice. Circulating triglyceride levels are thought to be regulated by the balance between its secretion and clearance. With lipoprotein catabolism suppressed, the increase in circulating triglycerides over time is indicative of the rate at which triglyceride is being secreted from the liver [84–86]. The hepatic triglyceride secretion rate was significantly lower in fenofibrate-treated mice when Triton WR1339 was used to prevent lipolysis. These observations suggest that the reduced circulating triglyceride levels after fenofibrate treatment are due to the decreased secretion of triglycerides from the liver. 

The molecular mechanisms underlying the effects of fenofibrate on obesity and lipid metabolism involve the changes in the expression of apolipoprotein C-III (apo C-III) and ACOX. LDLR-null mice fed fenofibrate showed significantly lower mRNA levels of hepatic apo C-III, an apolipoprotein that limits tissue triglyceride clearance [87, 88]. Fenofibrate-activated PPAR*α* in the liver increased mRNA levels of ACOX, the first and rate-limiting enzyme of PPAR*α*-mediated FA *β*-oxidation, which resulted in reduced triglyceride production [87]. 

In conclusion, fenofibrate prevents both obesity and hypertriglyceridemia through hepatic PPAR*α* activation in male LDLR-deficient mice.

### 3.3. Fenofibrate Regulates Female Obesity Depending on the Presence of Ovaries

Based on the suggestion that fenofibrate inhibits body weight gain and adiposity in male LDLR-null mice, it can be hypothesized that fenofibrate improves obesity in female LDLR-null mice. Body weight gain and WAT mass were significantly increased in both female OVX and sham-operated (Sham) LDLR-null mice on a high fat diet for 8 weeks. The increases in body weight and WAT mass were higher in female OVX LDLR-null mice than in Sham mice. Interestingly, fenofibrate-treated female OVX LDLR-null mice had lower body weights and WAT mass, similar to those found in several animal models, while female Sham mice did not exhibit these fenofibrate-induced reductions [21]. In *db*/*db* mice and fatty Zucker rats, the effect of fenofibrate on body weight depends on the utilization of FA, as demonstrated by a fenofibrate-induced increase of ACOX mRNA [9]. PPAR*α*-mediated FA *β*-oxidation and hydrolysis of triglycerides by fenofibrate contribute to decreased body weight and WAT mass in OVX LDLR-null mice, suggesting that fenofibrate can act as a body weight-regulator in an animal model of postmenopausal women. 

Serum triglycerides and total cholesterol were significantly increased in both female OVX and Sham LDLR-null mice. However, fenofibrate treatment substantially decreased high fat diet-induced increases of triglycerides and cholesterol in both female groups [9, 87]. In parallel with serum triglyceride levels, fenofibrate upregulated hepatic ACOX mRNA levels and downregulated apo C-III mRNA levels in both OVX and Sham LDLR-null mice [87, 88]. Such changes in mRNA levels of ACOX by fenofibrate were greater in female OVX LDLR-null mice than in Sham LDLR-null mice with functioning ovaries. 

However, it is not likely that the PPAR*α*-mediated reduction in serum triglycerides directly controls obesity in female Sham LDLR-null mice, which exhibited simultaneous decreases in serum triglycerides and increases in body weight and WAT mass. Thus, the effect of fenofibrate on the body weight of female Sham LDLR-null mice cannot be explained simply in terms of an altered and enhanced flux of FAs and triglycerides, since fenofibrate increased ACOX mRNA and decreased apo C-III gene expression in this group (although this expression was lower than in the OVX group). Moreover, these changes in ACOX and apo C-III mRNA did not correlate with increased body weight and adiposity. Such conflicting data suggest the possibility that this discordance may be caused by ovarian factors. 

**Figure 5:**
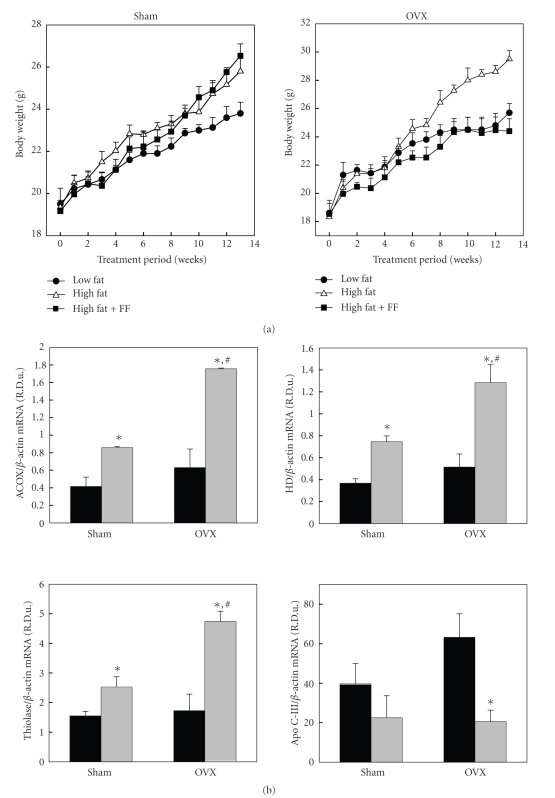
*Differential regulation of body weight gain (a) and PPAR* *α* *target gene expression (b) by fenofibrate depending on the presence of ovaries.* Female sham-operated (Sham) and ovariectomized (OVX) mice received a low fat, high fat, or fenofibrate-supplemented (FF; 0.05% w/w) high fat diet for 13 weeks. Body weights at the end of the treatment period are significantly different not only when comparing the low fat group to either the high fat (*P* < .05) or high fat plus FF (*P* < .01) groups in female Sham mice, but also when comparing the high fat group to either the low fat (*P* < .01) or high fat plus FF (*P* < .005) groups in female OVX mice. ∗: Significantly different versus the high fat group, *P* < .05. #: Significantly different versus the Sham group, *P* < .05. ACOX: acyl-CoA oxidase; HD: enoyl-CoA hydratase/3-hydroxyacyl-CoA dehydrogenase; thiolase: 3-ketoacyl-CoA thiolase; apo C-III: apolipoprotein C-III. Adapted from [23].

The regulation of obesity by fenofibrate in female wild-type C57BL/6J mice is similar to that in female LDLR-null mice. Fenofibrate reduced body weight gain and WAT mass in high fat diet-fed wild-type OVX mice but failed to do so in Sham mice (Figure 5(a)) [23]. Body weights of OVX mice were found to be higher than those of Sham mice 6 weeks after commencing the high fat diet. Compared to high fat diet-fed OVX mice, fenofibrate-treated OVX mice had significantly decreased body weight gain by 6 weeks into the treatment regimen and had significantly lower body weight at 13 weeks. In addition to changes in body weight, WAT mass was significantly reduced after fenofibrate treatment, and the final WAT mass of the fenofibrate-treated OVX animals was lower than that of the OVX animals on a regular chow diet. In contrast to the OVX mice, fenofibrate did not decrease body weight gain and WAT mass increases in Sham mice. These results suggest that obesity is differentially affected by fenofibrate treatment in Sham and OVX mice.

Fenofibrate reportably acts as a weight-stabilizer through PPAR*α* although these results were obtained using male animal models [9–11, 22]. Nevertheless, these reports suggest that fenofibrate not only prevents excessive weight gain but is also able to mobilize fat from adipose tissue by increasing fat catabolism in the liver. Notably, reductions in body weight gain and WAT mass by fenofibrate were similar in male and female OVX mice but were absent in female Sham mice. 

Fenofibrate seems to differentially affect body weight and adiposity among OVX and Sham mice by a mechanism other than the modulation of leptin gene expression. Although leptin is produced only in adipose tissue and elicits satiety responses by binding to leptin receptors in the brain [89, 90], changes in leptin mRNA levels are in accordance with those in body weight and WAT mass in both female OVX and Sham mice following fenofibrate treatment. Consistent with this finding, Guerre-Millo et al. [10] reported that serum leptin concentrations positively correlated with body weight and epididymal adipose tissue mass in fenofibrate-treated male mice [10], suggesting that fenofibrate modulates body weight, not by influencing leptin gene expression and food intake, but by enhancing energy expenditure [91, 92].

Differences in PPAR*α* target gene expression seem to explain the different effects of fenofibrate on gonad-dependent weight gain in females (Figure 5(b)). Fenofibrate not only elevated the transcriptional activation of PPAR*α* target genes, ACOX, HD, and thiolase but also reduced apo C-III mRNA levels compared to a high fat diet alone in both groups of mice. Moreover, these alterations in expression levels were found to be more prominent in female OVX mice than in Sham mice after fenofibrate treatment. Thus, fenofibrate influences obesity via the differential activation of PPAR*α*.

It has also been reported that ovarian steroids can affect obesity and lipid metabolism and that these effects are likely mediated by estrogens [12]. E2 insufficiency is thought to be largely responsible for increased adiposity and circulating lipids in OVX rodents because such animals do not display obesity, adiposity, and lipid disorders when they are administered E2 replacement [17–19]. Although the administration of E2 or fenofibrate alone effectively reduces body weight gain and WAT mass in high fat diet-fed female OVX mice, fenofibrate treatment does not prevent them in female Sham mice with functioning ovaries. These results suggest the possibility that signal cross-talk may exist between PPAR*α* and ERs in their effects on obesity and that the action of fenofibrate may be influenced by estrogens in females [25, 27, 93].

In conclusion, treatment with fenofibrate has different effects on body weight and WAT mass due in part to differentially activating hepatic *β*-oxidation and apo C-III gene expression between female Sham and OVX mice. These differences may provide important information about the mechanisms modulating obesity and about the actions of other lipid lowering drugs, such as fenofibrate, which are PPAR*α* ligands in females.

### 3.4. The Actions of PPAR*α* on Obesity Are Inhibited by Estrogens

My previous results show that the PPAR*α* ligand fenofibrate reduced body weight gain and adiposity in male and female OVX mice, but not in female mice with functioning ovaries [20–23], suggesting that the actions of fenofibrate on obesity are influenced by E2. 

E2 affects the ability of fenofibrate to reduce body weight gain and adiposity in female OVX mice. Mice fed a high fat diet with either fenofibrate or E2 for 13 weeks exhibited significant decreases in body weight gain and WAT mass compared to high fat diet-fed controls. These observations are supported by my previous results showing that fenofibrate stimulates hepatic FA *β*-oxidation in female OVX mice [21, 23], as well as by other reports showing that E2 inhibits feeding by decreasing meal size in OVX animals [94, 95]. However, these reductions were not enhanced when mice were concomitantly treated with fenofibrate and E2, indicating that E2 may inhibit the function of PPAR*α* in female obesity [32]. Evidence from both humans and laboratory animals show that E2 plays an important role in regulating body weight and WAT mass. Ovariectomy in rodents increases WAT mass, and E2 replacement decreases WAT mass [94]. Similarly, while postmenopausal women have increased body weight gain and WAT weight, E2 decreases both of these [96, 97]. Other studies have also suggested that fenofibrate reduces body weight gain in male animal models [9–11] but does not induce decreases in body weight and WAT mass gains in female mice [20, 21, 23], suggesting that E2 may inhibit the actions of fenofibrate on body weight and WAT mass in female OVX mice. 

Similarly, the combination of E2 and fenofibrate did not result in any additional beneficial effects on lipid metabolism in female OVX mice. While serum levels of total cholesterol and triglycerides were lowered in mice fed a high fat diet with either fenofibrate or E2 compared with mice fed a high fat diet alone [9, 18], the combination of E2 and fenofibrate increased levels of circulating total cholesterol and triglycerides compared with either E2 or fenofibrate alone. These results are in agreement with findings that the combination of a lipid-lowering fibrate and hormone replacement therapy (HRT) for 3 months not only had no additional benefits on the routine serum lipid or lipoprotein profiles in overweight postmenopausal women with elevated triglycerides but also increased serum triglycerides [97]. Consistent with the circulating lipid metabolism, the fenofibrate-induced decrease in hepatic lipid accumulation was also increased by E2 in female OVX mice. Mice fed a high fat diet showed considerable hepatic lipid accumulation, which was prevented by fenofibrate or E2. In contrast, mice concomitantly treated with fenofibrate and E2 showed an accumulation of triglyceride droplets. Thus, it appears that E2 inhibits fenofibrate-induced increases in fat catabolism in the liver of female OVX mice. Fenofibrate-treated OVX mice were found to have similar food intake to Sham controls whereas OVX mice given E2 showed decreased food intake. However, a combinational treatment of fenofibrate and E2 increased body weight gain, fat weight, and hepatic fat accumulation compared with fenofibrate alone, despite similar food consumption profiles between E2 and fenofibrate plus E2 groups, suggesting that E2 may affect the ability of fenofibrate to regulate energy balance. 

Fenofibrate-activated PPAR*α* has been shown to regulate the expression of a number of genes critical for FA *β*-oxidation and lipid catabolism. Fenofibrate upregulated ACOX, HD, and thiolase mRNA levels whereas E2 downregulated the transcriptional activation of these genes. Coadministration of fenofibrate and E2 significantly decreased ACOX, HD, and thiolase mRNA levels compared with fenofibrate treatment. These results were in accordance with serum levels of triglycerides and total cholesterol as well as body weight and WAT mass. Thus, inhibition of the actions of PPAR*α* on body weight, WAT mass, and circulating lipid levels by E2 may be attributed, in part, to reductions in hepatic mRNA expression of PPAR*α*-mediated peroxisomal FA *β*-oxidizing enzymes by E2. 

**Figure 6:**
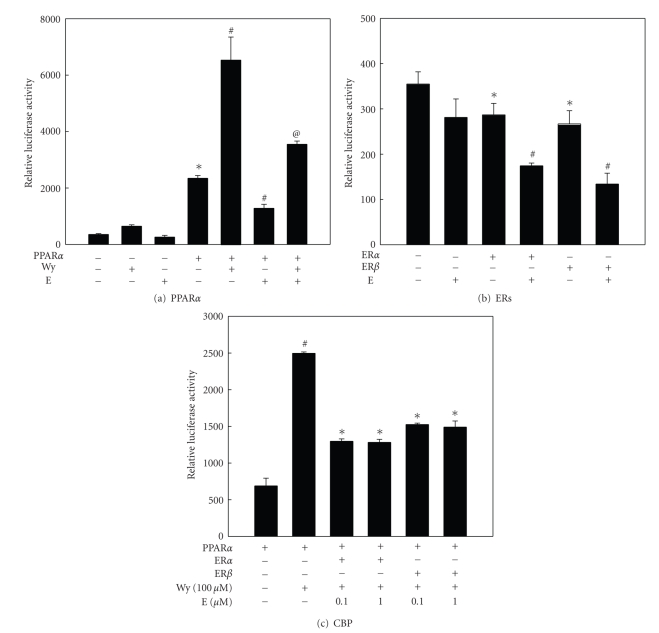
*Inhibition of PPAR*α* reporter gene expression ((a) and (b)) and coactivator recruitment (c) by 17* *β* *-estradiol.* (a) NMu2Li cells were transiently transfected with expression plasmids for PPAR*α* and PPRE_3_-TK-Luc reporter. * Significantly different versus control group, *P* < .0001. #: Significantly different versus PPAR*α* group *P* < .0001. @ Significantly different versus PPAR*α*/Wy group, *P* < .001. (b) NMu2Li cells were transiently transfected with expression plasmids for PPRE_3_-TK-Luc reporter and ER*α* or ER*β*.∗: Significantly different versus control group, *P* < .05.#: Significantly different versus respective ER group, *P* < .01. (c) CV-1 cells were transiently transfected with expression plasmids for VP16-mPPAR*α*, GAL-CBP, reporter plasmid pFR-Luc, and VP16-hER*α* or VP16-hER*β*. #: Significantly different versus PPAR*α* group, *P* < .01.∗: Significantly different versus PPAR*α*/Wy group, *P* < .005. Adapted from [32].

Consistent with the in vivo data, E2 inhibited basal PPAR*α* reporter gene activity as well as Wy14,643-induced reporter gene activation in NMu2Li murine liver cells transfected with PPAR*α*, showing that E2 can modulate PPAR*α* transactivation (Figure 6(a)). The inhibitory activity by E2 is mediated through its binding to endogenous ERs that are normally expressed in NMu2Li liver cells since it is reported that E2 does not bind directly PPARs [98]. However, the possibility that E2 directly binds to PPAR*α* and inhibits PPAR*α* function cannot be excluded, because no binding studies have been performed. In cells transfected with either ER*α* or ER*β*, ERs inhibited the basal expression of PPRE-mediated reporter gene activity (Figure 6(b)). These inhibitory effects were significantly increased by E2 treatment. This is supported by results showing that PPARs can regulate ER target gene expression and that signal cross-talk between ERs and PPARs has been reported to be bidirectional [24–26, 28, 93]. 

Mechanistic studies revealed that the E2-ER complex was not likely to be competent for PPAR*α* transactivation, as indicated by the inability of E2 to stimulate PPAR*α* recruitment of coactivators such as CBP (Figure 6(c)). Ligand-induced conformational changes that allow recruitment of coactivators, such as CBP and the dissociation of corepressors such as NCoR, are obligatory for transactivation by PPAR*α*. Treatment of transfected CV-1 cells with Wy14,643 caused efficient CBP recruitment as evidenced by an increase in luciferase reporter gene activity. However, E2 significantly decreased Wy14,643-induced CBP association in the presence of ER*α* or ER*β*. Thus, inhibition of PPAR*α* transactivation by ERs was due to competition for coactivators, increased availability of corepressors, or some other mechanism. [26, 28] It has previously been shown that competition of distinct nuclear receptor for coactivator binding results in a negative cross-talk between nuclear receptors [99, 100]. These results suggest that E2 inhibition of PPAR*α* function occurs by impairing the recruitment of transcriptional coactivators. 

PPAR*α* and ERs bind to short DNA sequences termed HREs, ERE for ERs and PPRE for PPAR*α* [54, 101]. An ERE is an inverted repeat containing three intervening bases (AGGTCA N_3_ TGACCT) whereas a PPRE is a direct repeat with one or two intervening sequences (AGGTCA N_1,2_ AGGTCA). Nonetheless, these sequences contain an AGGTCA half site, which could be recognized by either ERs or PPAR*α*. Signal cross-talk between PPAR/RXR and ERs has been reported to occur through competitive binding to ERE [24]. Therefore, the inhibition of PPAR*α* transactivation by ERs may also have been due to their competition for PPRE. 

**Figure 7:**
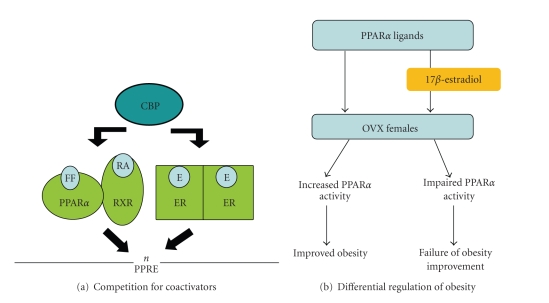
*Mechanism of inhibitory effect of *17*β*-*estradiol on PPAR* *α* *-mediated regulation of obesity.* (a) Competition between PPAR*α* and estrogen receptors (ERs) for coactivator binding. 17*β*-estradiol-activated ERs can interfere with the PPRE binding of PPAR*α*. (b) Inhibition of PPAR*α* actions on obesity by E. E impairs the ability of PPAR*α* ligands to reduce body weight gain and adiposity in female ovariectomized (OVX) mice. FF: fenofibrate; RA: 9 cis-retinoic acid; RXR: retinoid X receptor. Adapted from [33].

 In conclusion, in vivo and in vitro studies demonstrate that E2 inhibits the actions of PPAR*α* on obesity through its effects on hepatic PPAR*α* -dependent regulation of target genes and that these processes are mediated by inhibition of PPAR*α* recruitment of coactivators by E2-activated ERs (Figure 7). PPAR*α* ligands fibrates may act as efficient weight controllers under estrogen-free conditions. Although E2 alone decreases body weight gain and WAT mass, E2 may impair PPAR*α* actions on obesity. Thus, these results provide a rationale for the use of fenofibrate in men and postmenopausal women with obesity and lipid disorder, but not for premenopausal women with functioning ovaries.

## 4. Conclusion

Obesity is the leading cause of the metabolic diseases including type 2 diabetes, atherosclerosis, and hypertension. PPAR*α* has been the subject of intense academic and pharmaceutical research because of its ability to improve obesity-related metabolic disorders. The PPAR*α* ligand fenofibrate seems to exhibit an antiobesity effect through FA *β*-oxidation in animal models although such an effect of PPAR*α* activators has not yet been reported in humans. However, this idea is supported by several human studies showing that obese patients with impaired fat oxidation failed to lose weight, suggesting that elevated fat oxidation leads to weight loss. Interestingly, there is a sex difference in the control of obesity by fenofibrate. Fenofibrate regulates body weight and adiposity with sexual dimorphism in nutritionally induced obese male mice. Moreover, fenofibrate-induced reductions in body weight gain and WAT mass in male mice were also shown by female OVX mice, but these effects were absent in female Sham mice, suggesting the involvement of ovarian hormones in the differential regulation of obesity among these groups. In OVX mice, E2 inhibited the actions of fenofibrate-activated PPAR*α* on obesity, due in part to reductions in hepatic expression of PPAR*α*-mediated FA *β*-oxidizing enzymes by E2, a process mediated through the inhibition of PPAR*α* coactivator recruitment by E2. These results provide a mechanism to explain why fenofibrate reduces body weight gain and adiposity in males and OVX female mice but does not regulate obesity in female mice with functioning ovaries.

## Figures and Tables

**Table 1:** PPAR*α* target genes involved in lipid homeostasis.

Target genes	Gene expression
Fatty acid uptake, binding, and activation	
Fatty acid transport protein (FATP)	Stimulation
Fatty acid translocase (FAT/CD36)	Stimulation
Liver cytosolic fatty acid-binding protein (L-FABP)	Stimulation
Acyl-CoA synthetase (ACS)	Stimulation
Carnitine palmitoyltransferase I and II (CPT-1and CPT-II)	Stimulation
Mitochondrial fatty acid *β*-oxidation	
Very long-chain acyl-CoA dehydrogenase (VLCAD)	Stimulation
Long chain acyl-CoA dehydrogenase (LCAD)	Stimulation
Medium-chain acyl-CoA dehydrogenase (MCAD)	Stimulation
Short-chain acyl-CoA dehydrogenase (SCAD)	Stimulation
Peroxisomal fatty acid *β*-oxidation	
Acyl-CoA oxidase (ACOX)	Stimulation
Bifunctional enzyme (HD)	Stimulation
3-Ketoacyl-CoA thiolase (Thiolase)	Stimulation
Hydrolysis of plasma triglycerides	
lipoprotein lipase (LPL)	Stimulation
Apolipoprotein C-III (Apo C-III)	Inhibition
Fatty acid synthesis	
Acetyl-CoA carboxylase (ACC)	Inhibition
Fatty acid synthase (FAS)	Inhibition
HDL metabolism	
Apolipoprotein A-I and A-II (ApoA-I and ApoA-II)	Stimulation
ATP-binding cassette transporter 1 (ABCA1)	Stimulation
Electron transport chain	
Uncoupling protein 1, 2, and 3 (UCP1, 2, and 3)	Stimulation
